# Spontaneous Charging from Sliding Water Drops Determines the Interfacial Deposition of Charged Solutes

**DOI:** 10.1002/adma.202420263

**Published:** 2025-03-07

**Authors:** Xiaoteng Zhou, Yuwen Ji, Zhongyuan Ni, Javier Garcia Lopez, Kalina Peneva, Shan Jiang, Nikolaus Knorr, Rüdiger Berger, Kaloian Koynov, Hans‐Jürgen Butt

**Affiliations:** ^1^ Max Planck Institute for Polymer Research Ackermannweg 10 55128 Mainz Germany; ^2^ Institute of Organic Chemistry and Macromolecular Chemistry Friedrich Schiller University Jena Lessingstraße 8 07743 Jena Germany; ^3^ Center for Energy and Environmental Chemistry Jena (CEEC Jena) Friedrich Schiller University Jena Philosophenweg 7a 07743 Jena Germany; ^4^ Jena Center for Soft Matter (JCSM) Friedrich Schiller University Jena Philosophenweg 7a 07743 Jena Germany; ^5^ Department of Materials Science and Engineering Iowa State University of Science and Technology Ames IA 50011 USA; ^6^ Department of Mechanical Engineering Massachusetts Institute of Technology 77 Massachusetts Avenue Cambridge MA 02139 USA

**Keywords:** interfacial phenomena, mass transfer, slide electrification, ssDNA, wetting

## Abstract

It has been discovered during the last decade that when water drops slide on hydrophobic surfaces, they spontaneously leave negative charges along the drop path. The drops become positively charged with a potential of 1 kV. This process, called *slide electrification*, influences drop motion and alters contact angles. Here, a third effect of slide electrification is demonstrated: the preferential deposition of dissolved solutes with positive charges. To illustrate this, water drops containing dissolved charged fluorophore ions are allowed to slide down a tilted hydrophobic surface, and their track is imaged. Two perylene derivatives are applied as fluorophores, one chromophore carrying positive charges, PDI^+^, and one carrying negative charges, PDI^─^. PDI^+^ is deposited at a concentration as low as 0.5 µm. In contrast, PDI^─^ is only deposited above 5 µm. Experiments using grounded drops or a hydrophobic coating on a conducting substrate indicate that the electric field generated from the negative surface charges behind the drop causes a preferential deposition of the dissolved ions near the interface. This hypothesis also agrees with Kelvin probe measurements. Complex biomolecules deposition e.g. DNA can be also affected by this. These findings contribute to a better understanding of mass transfer processes at interfaces.

## Graphical abstract

When water drops slide on hydrophobic surfaces, they spontaneously leave behind negative charges along their path, resulting in the drops becoming positively charged. Here, it is shown that this phenomenon, known as slide electrification, influences the deposition of charged dissolved molecules. Using charged fluorophores and biomolecules, a preferential deposition of cations along the drop's trail is demonstrated, following the charge distribution pattern.

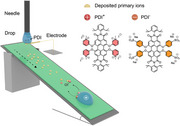

## 1 Introduction

Moving water drops are a common phenomenon in daily life.^[^ ^1^ ^]^ This occurrence is significant in various industrial applications including cooling,^[^ ^2^ ^]^ extraction and separation,^[^ ^3^ ^]^ water desalination,^[^ ^4^ ^]^ and microfluidics.^[^ ^5^ ^]^ In recent years, it has been realized that when a water drop slides on^[^ ^6^ ^]^ or impacts^[^ ^7^ ^]^ a hydrophobic surface, it spontaneously deposits negative charges on the substrate.^[^ ^8^ ^]^ As a result, the drop itself gets positively charged. The potential between the drop and deposited charges can reach more than 1000 V.^[^ ^9^ ^]^ This phenomenon is called *slid* *e* or *contact electrification*.^[^ ^10^ ^]^

Many experiments have shown that the charges deposited originate from the surface charges formed spontaneously when the solid surface is contacted by an electrolyte, especially water.^[^ ^10^, ^11^ ^]^ When a solid surface gets into contact with water, an electric double layer (EDL) is formed through the spontaneous deposition of ions or the dissociation of surface groups.^[^ ^12^ ^]^ The electric double layer is composed of bound surface charges and a diffuse layer of counter charges in the liquid drop.^[^ ^13^ ^]^ When a water drop slides off the surface, the surface acquires a bound net charge due to its surface chemistry.^[^ ^11^ ^]^ For most hydrophobic surfaces, the surface charge is reported to be negative,^[^ ^6^, ^14^ ^]^ often attributed to an enrichment of ions.^[^ ^14^ ^]^ The ions partially remain on the solid surface and are separated from the diffuse part of the EDL at the receding contact line.^[^ ^11^ ^]^

To date, a major consequence of drop charging has been extensively analyzed: the influence on drop motion.^[^ ^6^, ^7^, ^15^ ^]^ Spontaneous charging of drops retards their motion and changes the contact angles. Here, we explore another significant consequence: the selective deposition of dissolved, charged solutes. Electrocoating of metal surfaces is an established industrial process.^[^ ^16^ ^]^ In this process, a potential is applied to a metal immersed in a suspension of oppositely charged paint solutes. The paint particles are then deposited on the surface to form a film. Recently, Knorr et al. also visualized particle deposition on water pre‐charged insulating surfaces by applying and removing charged toners.^[^ ^17^ ^]^ They observed strong variations on the sub mm scale. Here we explore whether spontaneous charging on insulting surfaces can lead to a synchronous solute deposition during this process. To our knowledge, there has been no systematic study of the influence of spontaneous drop charging on the deposition of dissolved, charged molecules. Due to the self‐ionization of water, water drops always contain hydroxyl (OH^─^) and hydronium (H_3_O^+^) ions. Ions coming from dissolved salts,^[^ ^14^ ^]^ such as NaCl or KNO_3_,^[^ ^18^ ^]^ or dissolved CO_2_ reacting to H_2_CO_3_ (↔ HCO_3_^─^ + H^+^) are also common components in natural water.^[^ ^19^ ^]^ In the following, we refer to these potential‐determining ions that dominate the process of common slide electrification^[^ ^11^ ^]^ as *primary ions*. Here, we examine deposition using drops containing additional larger dissolved molecules that become charged due to dissociation or uptake of an ion.^[^ ^20^ ^]^ These additionally dissolved, larger charged molecules are called *secondary ions*.

The nature of the *primary ions* and charge formation at the water‐hydrophobic interface is not yet clear,^[^ ^21^ ^]^ thus we talk about *primary ions* in the following without specifying their chemical nature and the deposition. Negative zeta potentials have been observed for water‐oil,^[^ ^22^ ^]^ water‐air,^[^ ^23^ ^]^ and potentiometric titration measured water‐solid hydrophobic interfaces.^[^ ^24^ ^]^ The pH dependence of the zeta potential and the fact that most anions do not have a strong effect on the zeta potential indicates that enrichment of hydroxyl may cause a negative interfacial potential.^[^ ^25^ ^]^ However, sum frequency generation (SFG) spectroscopy^[^ ^26^ ^]^ and resonant UV second‐harmonic generation (SHG) experiments^[^ ^21^, ^27^ ^]^ do not find such a surface excess of OH^─^. Some simulations indicate high energy barriers of hydroxyl adsorption for water‐oil and water‐air interfaces,^[^ ^26^, ^28^ ^]^ and for solid hydrophobic surface in contact with water a weak enrichment of OH^─^ was obtained by molecular dynamic simulations.^[^ ^29^ ^]^ There is an ongoing debate about the primary potential‐determining ions at interfaces, possibly involving specific formations from hydroxyl groups or electrons within water molecules, which is not the scope of our focus here.

**Figure 1:**
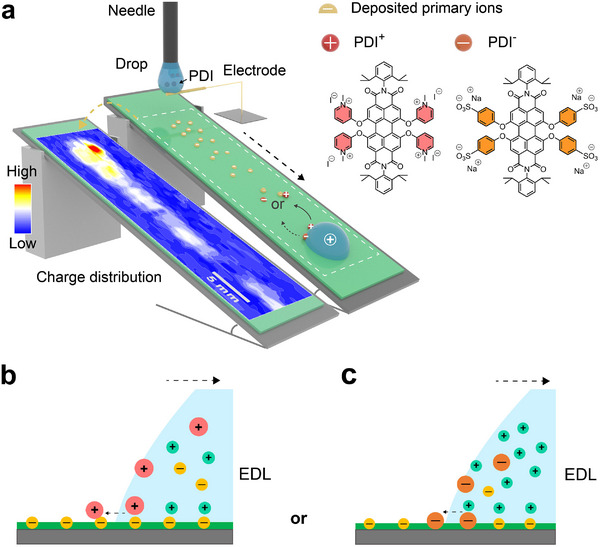
Experimental design. a) Schematic of the experimental setup: a water drop slides down an inclined glass slide coated with a hydrophobic layer. Due to the process of slide electrification negative primary ions with varying density are left on the surface behind the drop as illustrated schematically (right) and experimentally (Section S1, Supporting Information) by Kelvin probe measurements (left). For water drops containing charged PDI dyes we outline possible mechanisms for PDI^+^ b) and PDI^─^ c) deposition during drop sliding. The red and orange dots represent the secondary ions PDI^+^ and PDI^─^. The yellow dots represent the small primary ions and their counterions (light green). The hydrophobic coating is indicated in dark green.

The key questions we address here are: Does the high potential in the drop affect the deposition of the charged *secondary ions* (**Figure** **1**)? In particular, does it lead to an enhanced deposition of positively charged *secondary ions* by electrostatic attraction to the *primary* ions adsorbed on the surface (Figure 1b)? Alternatively, do negatively charged *secondary ions* deposit together with the *primary* ions and enhance the charge separation (Figure 1c)?

To answer these questions, we used water‐soluble fluorescent dyes that serve as secondary ions to visualize the deposition. Using a sensitive imaging system, fluorescence allows us to detect even tiny amounts of deposited solutes. We used N,N′‐bis(2,6‐diisopropylphenyl)‐1,6,7,12‐tetra‐[3‐(N‐methylpyridinium)oxy] perylene‐3,4,9,10‐tetracarboxylic acid diimide iodide (PDI^+^) as a cationic fluorescent dye and N,N′‐bis(2,6‐diisopropylphenyl)‐1,6,7,12‐tetra‐[(4‐sulfonyl)phenoxy] perylene‐3,4,9,10‐tetracarboxylic acid diimide sodium (PDI^─^) as an anionic one. Both dyes share the same fluorophore scaffold but have differently charged side groups (Figure 1). They are chemically and photochemically stable and have a high quantum yield in water.^[^ ^30^ ^]^ Drops of water containing dissolved PDI dyes were pipetted onto hydrophobic surfaces tilted at 40°. Afterward, the surfaces were imaged with laser scanning confocal microscopy (LSCM) along the drop path in 2 dimensions. We demonstrated that the drop slide electrification led to preferential deposition of cationic PDI^+^ over anionic PDI^─^. This new finding contributes to our understanding of mass transfer in chemical and mechanical engineering.^[^ ^3^ ^]^ Understanding molecule deposition behind sliding drops is crucial for applications such as coating vehicles, aircraft, solar cells,^[^ ^31^ ^]^ and sensors.^[^ ^32^ ^]^ Furthermore, we found that the deposition of complex biological molecules, such as fluorescent single‐stranded DNA (ssDNA) with positive end groups, is also affected by slide electrification. Therefore, the effects described in this manuscript could potentially help to achieve a better understanding of the selective deposition of biomolecules^[^ ^33^ ^]^ by electrostatic interaction and realize low‐cost biosensing chip fabrication by electrodepositing biomolecules, e.g., DNA.^[^ ^34^ ^]^

## 2 Results and Discussion

**Figure 2:**
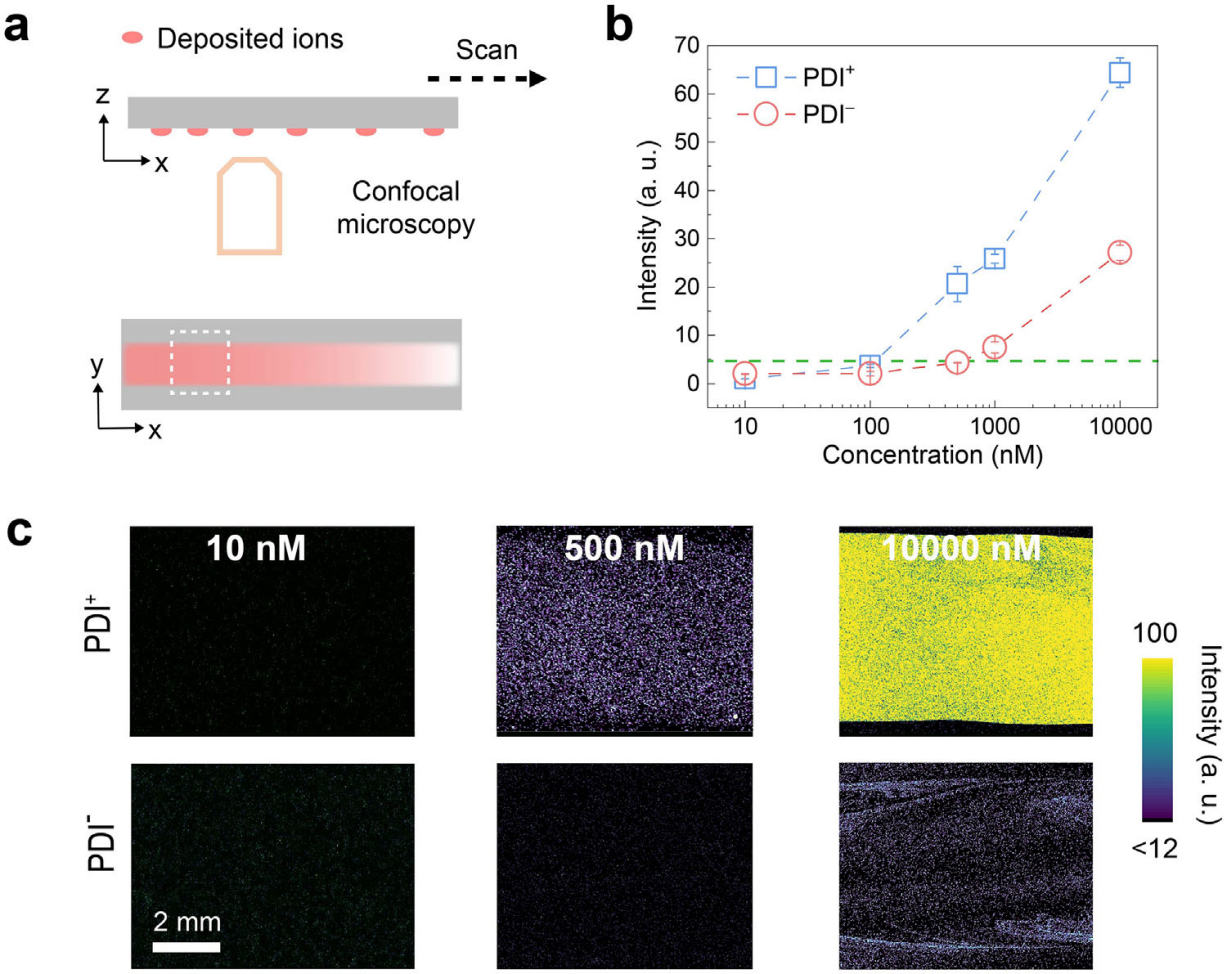
Deposition of PDI^+^ and PDI^−^ after one drop sliding. a) Schematic showing how deposited dyes were imaged by laser scanning confocal microscopy (top, x‐z) after a dyed drop slid over the surface and how the drop path appears from such a scan (bottom, x‐y). b) The average fluorescence intensities obtained from the images in the position shown in (a) versus the PDI concentrations in the sliding drop. c) Laser scanning confocal microscopy images of the initial drop path (6 mm away from the first contact position) recorded after the sliding of one drop containing either PDI^+^ or PDI^−^ on PFOTS‐coated glass, with increasing concentrations.

Single water drops containing either PDI^+^ or PDI^−^ were allowed to slide down on thin glass slides functionalized with perfluorodecyltrichlorosilane (PFOTS). By using confocal microscopy, the deposition of both PDI^+^ and PDI^−^ was found to be concentration‐dependent, with PDI^+^ depositing significantly more than PDI^−^ at lower concentrations (**Figure** **2**). Below a concentration of 0.1 µm deposits of neither PDI^+^ nor PDI^−^ were detected. The fluorescence signal, if any, could not be distinguished from the background noise (Figure 2b,c). When the concentration was increased to 0.5 µm, PDI^+^ was deposited, but PDI^−^ was not. At 1 µm, the same was observed. Only when using drops with 10 µm concentration were both dyes deposited. However, the fluorescence signal from deposited PDI^−^ was three times lower than that from PDI^+^ and showed an increased uneven distribution for the same sliding distance. The excitation laser light intensities used for the LSCM imaging of the PDI^+^ and PDI^−^ were calibrated in a way that similar fluorescence intensities were detected from surfaces covered with the same amounts of PDI^+^ or PDI^−^ (Experimental Section and Section S2, Supporting Information). Therefore, we concluded that a much smaller amount of PDI^−^ is deposited as compared to PDI^+^ at such a concentration range (10 nm to 10 µm). An ≈10‐fold higher concentration of PDI^−^ is required to achieve the same fluorescence and deposition density as with PDI^+^. If we attribute this to a difference in adsorption energy via the Boltzmann factor, exp(Δ*E*/RT)=  10, the energy difference Δ*E* would be 2.3RT = 5.5 J mol^−1^, where R = 8.3 J K^−1^ mol^−1^ and T is the absolute temperature.

These results indicate that positively charged molecules are preferentially deposited (Figure 1b), while negatively charged ones are not. The scenario illustrated in Figure 1c is not consistent with our observations for a concentration ≤1 µm. Furthermore, neither the pH value nor the surface tension changed substantially when adding either PDI^+^ or PDI^−^ molecules to water. The pH value remained ≈6.5 at least up to a concentration of 10 µm. The surface tension measured with a Wilhelmy plate remained between 70 and 72 mN m^−1^. We also concluded that adding PDI dyes does not change the drop properties, indicating that only the surface property changes influence deposition.

Based on the LSCM results and measurements of drop properties, we can further assume that the strong difference in the deposition of positively and negatively charged dyes was caused by electrostatic interactions. Indeed, when a water drop moves down a PFOTS‐coated glass slide, it usually acquires a charge of +1.2 to 1.6 nC.^[^ ^6^, ^9^ ^]^ Negative *primary ions* charge the surface behind the drop. The surface charge density is of the order of 15 µC m^−2^.^[^ ^10^ ^]^ The drop charge leads to a drop potential that can be estimated from the capacitance *C* of the drop and the dielectric permittivity of the substrate. With a glass substrate of *d* ≈ 1 mm thickness, and a permittivity of *ε* = 5, the capacitance of the sample is *C*  = *A*εε_0_/ d . Here, ε_0_ = 8.85×10^−12^ C^2^N^−1^m^−2^ and *A* is the contact area of the drop. With *A* ≈ 27 mm^2^ we get a capacitance of *C* ≈ 1.2 pC. A drop charge of *Q* = 1.2 nC leads to a drop potential U  = *Q*/*C*  of the order of 1000 V as reported.^[^ ^9^ ^]^ The resulting high field strength is high enough to promote deposition of positively charged solutes (*secondary ions*) like PDI^+^ and repel negatively charged *secondary ions* like PDI^−^. The observation that PDI^−^ is also deposited, although only when it is present at very high concentration in the drop suggests that there are other interactions besides electrostatic effects. Since the scaffold of the molecule is hydrophobic, we attribute the deposition to hydrophobic interactions. Due to this, at a high concentration PDI^−^ starts to deposit despite the electrostatic repulsive force, but the amount is much smaller than that of PDI^+^, where deposition is enhanced by the electrostatic attractive force.

**Figure 3:**
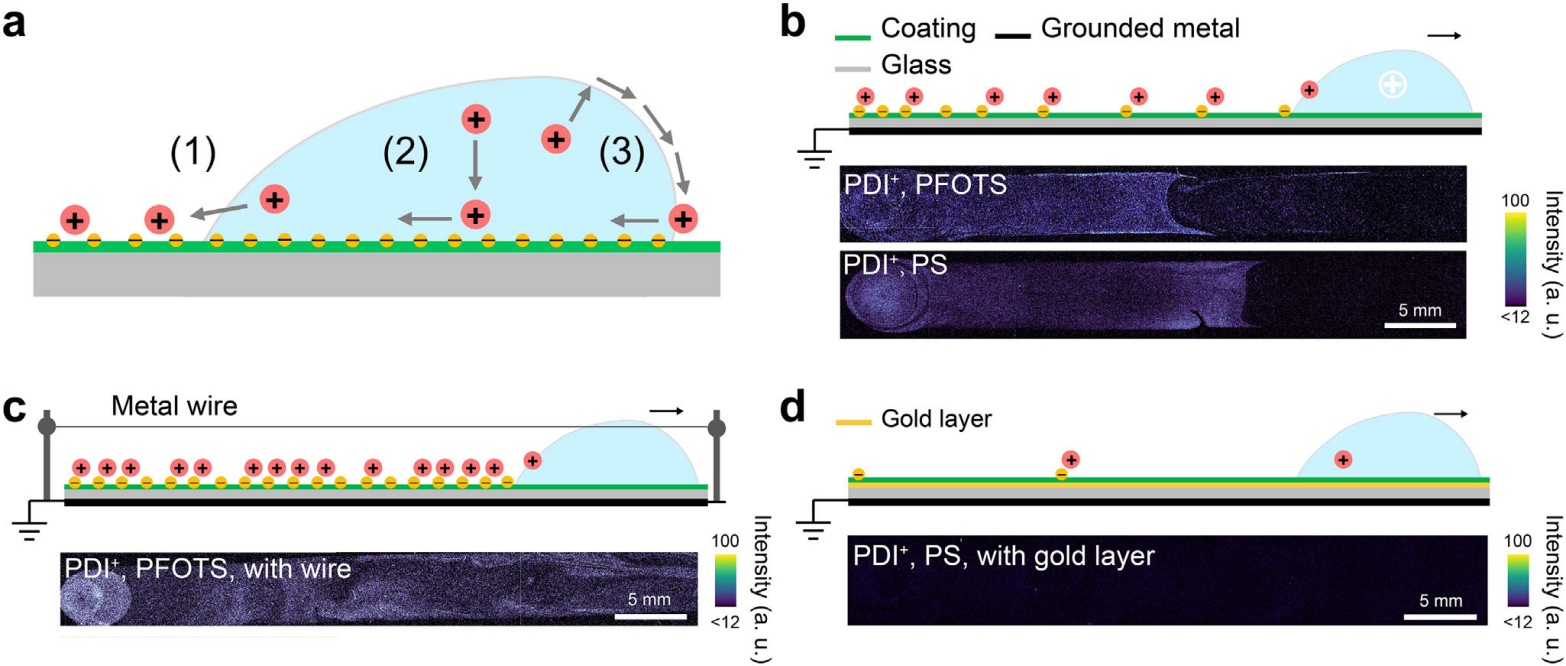
The deposition mechanism. a) Schematic illustration of three possible deposition processes of PDI^+^ (red dots) due to slide electrification. The *primary anions* are depicted as yellow dots. b) Schematic illustration of the PDI^+^ deposition on the drop path and the corresponding composite fluorescence images obtained for drops sliding on PFOTS or PS coated on glass. c) Schematic illustration and the corresponding composite fluorescence image for the PDI^+^ deposition on the drop path on PFOTS‐coated glass when the drop was grounded by a metal wire. d) Schematic illustration and the corresponding composite fluorescence image for the PDI^+^ deposition on the drop path on PS surface with a gold layer underneath. In (b–d) drops containing PDI^+^ with concentrations of 500 nm were used.

To elucidate the exact mechanism causing the preferred deposition of *secondary cations* like PDI^+^, we considered three possible processes (**Figure** **3**a):

1) The electric field emanating from the deposited *primary anions* near the receding contact line drives the *secondary cations* to co‐adsorb to the surface. They bind to the solid surface near the receding contact line when the drop slides off.

2) The *secondary ions* are already attracted to the solid‐liquid interface. The *secondary cations* remain on the surface when the rear contact line moves over them.

3) Like many organic molecules, PDI^+^ is partially hydrophobic and surface active. It tends to move to the water‐air surface during sliding and adsorb.^[^ ^35^, ^36^ ^]^ Such adsorption may also be enhanced by the positive potential of the drop. Due to the rolling component of hydrodynamic flow in the drop, the *secondary cations* could be deposited at the front contact line and remain adsorbed even when the drop passes over it.

In each of these assumptions, we use the term “adsorption” when the binding to an interface is dynamically reversible – at least in principle. Once a larger (nonvolatile) molecule is bound to a *free* solid surface, we call it “deposited”. It cannot spontaneously unbind.

To decide between these three possibilities, we analyzed the distribution of deposited PDI^+^ on the sliding drop path (Figure 3b). PDI^+^ is preferentially deposited during the first ≈2.5 cm of its slide path. Then its surface density decreases. This decrease was observed to be abrupt, which may indicate the deposition needs to overcome a certain energy barrier.^[^ ^37^ ^]^ A meniscus present before the end of deposition may result from non‐uniform charge deposition perpendicular to the drop's sliding direction, causing deposition to conclude later at the edges which will be further investigated in our future work. A similar distribution of PDI^+^ was observed on 20 nm thick polystyrene (PS) layer^[^ ^6^ ^]^ (Figure 3b). Experiments were all carried out at a relative humidity of 30 ± 5% and a temperature of 25 ± 0.5 °C. Variation in humidity (10% and 80%) does not affect the overall trend of deposition patterns but leads to a higher inhomogeneity (Figure S4, Supporting Information). Altering the temperature will change the surface energy, resulting in unstable results, so the experiments should be carried out in a chamber hood with controlled temperature and humidity.

The overall trend of decreasing concentration of PDI^+^ correlates with the decreasing charge density behind a water drop as measured by a Kelvin probe (Figure 1a; Section S1, Supporting Information). Assuming that the charge density on the free solid surface σ_S_ is related to the charge density of the solid‐liquid interface σ_L_ by σ_S_ = α σ_L,_ the charge density on the surface decays exponentially:^[^ ^9^, ^10^ ^]^

(1) $$\sigma_{s}=\alpha \sigma_{L}e^{-\chi /\lambda }$$

here, α is the transfer coefficient at zero drop potential, which may be related to drop velocity,^[^ ^11^ ^]^ humidity, etc., and *x* is the distance along the drop path. As a result, at the beginning of the slide path the charge density of the substrate is high. Then it decreases with a characteristic decay length λ of the order of 10 mm.^[^ ^10^, ^11^ ^]^ After long slide distances, charge deposition stops, and the drop reaches a steady state potential. This saturation behavior results in a highly charged drop path of ≈2 cm. Thus, the amount of deposited PDI^+^ correlates with the density of deposited primary anions behind the drop.

These observations seem to be in line with deposition process (1) in Figure 3: *secondary ions* are moved to the rear contact line. The electrostatic interaction at the microscale contact line may govern deposition, correlating with surface charge density changes (Figure 1a; Section S1, Supporting Information). To further support this hypothesis, we grounded the drop with a thin metal wire as reported before (Figure 3c).^[^ ^9^, ^15^ ^]^ By grounding the drop, its potential remains zero during the sliding. Consequently, the drop keeps depositing *primary anions*, and the charge separation will not attenuate with the sliding distance (scheme in Figure 3c). With respect to the deposition of PDI^+^, we observed that a grounded drop deposited the dye homogeneously over the whole slide path (Figure 3c), as expected. We conclude that the deposition of PDI^+^ is not caused by the overall positive potential of the drop as the process (3) in Figure 3a, but rather by the negative surface charge deposited behind the three‐phase contact line due to the slide electrification (process (1) in Figure 3a).

As an additional control, we carried out experiments on a 20 nm polystyrene (PS) film with a gold layer between it and the glass substrate. Unlike PFOTS, PS is a common homogenous coating that can fully cover the gold interlayer by dip‐coating. Such a conductive gold interlayer was demonstrated to prevent drop charging during sliding, screen possible deposited primary charges and lead to fast discharging.^[^ ^6^ ^]^ In our experiment, much less deposition of PDI^+^ was detected on the PS‐gold surface after a water drop with 0.5 µm PDI^+^ slid off due to the highly shield charge separation (Figure 3d). In contrast, PDI^+^ was obviously deposited on a PS film on glass without the gold interlayer (Figure 3b). Similar deposition prevention was observed on a PFOTS‐coated surface with an oxidized aluminum inter layer underneath (Section S4, Supporting Information). This demonstrates the generality of the phenomenon and shows that it can be potentially applied for creating patterned deposition adding yet another perspective to the broad subject of using charged surfaces to manipulate droplets and their interactions.^[^ ^38^ ^]^ We conclude that the deposition of positively charged solutes is dominated by slide electrification and not by direct adsorption to the solid‐liquid interface (process 2 in Figure 3a). All three experiments – distribution of deposited PDI^+^, grounded drop, and conductive substrate – indicate that process (1) is the most likely deposition process. Moreover, when pre‐charging the surface by sliding 10 pure water drops, the deposition from a drop containing 500 nm PDI^+^ slightly increased compared to a drop sliding on a non‐charged surface, further supporting this conclusion (Section S5, Supporting Information).

To estimate the surface density of deposited *secondary cations*, we considered the reduction in drop charge. If the PDI^+^ is deposited due to attraction from the negative surface charges behind the rear contact line, the drop charge should be substantially reduced. This was indeed the case. The charge of a pure water drop was +1.2 nC after sliding 4 cm on a PFOTS surface. With 500 nm PDI^+^ the drop charge was only +0.6 ± 0.2 nC after sliding the same distance. Roughly 50% of the deposited charge acquired during sliding due to *primary anions* is compensated for by the deposition of secondary cations PDI^+^. Similarly, drops containing 500 nm of another positively charged dye, Rhodamine B, charged to only +0.4 ± 0.2 nC. From these numbers we can estimate the number of deposited secondary ions. Assuming that this reduction in drop charge is due to PDI^+^ deposition behind the drop, the number of deposited PDI^+^ can be calculated as:

(2) n=ΔQ/eZ

where *e* is the elementary charge of 1.60×10^−19^ C and *Z* is the valency of one dye molecule at neutral pH. Considering *Z* = 4 according to the charge amount per molecule and assuming most of the molecules dissociated,^[^ ^30^ ^]^ and assuming that the dye molecules are evenly distributed over an area of say 20 × 5 mm^2^, this would lead to an average surface density of 1.7 × 10^13^ molecules m^−2^. Similarly, we estimated average density of 2.1 × 10^13^ and 1.4 × 10^13^ molecules m^−2^ for PS and polydimethylsiloxane (PDMS), respectively. Thus, the possible mean spacing *d* between the PDI^+^ molecules on the studied surface would be between 200  and 300 nm. These values are consistent with an estimation based on complementary fluorescence correlation spectroscopy^[^ ^39^ ^]^ measurements (Section S6, Supporting Information).

**Figure 4:**
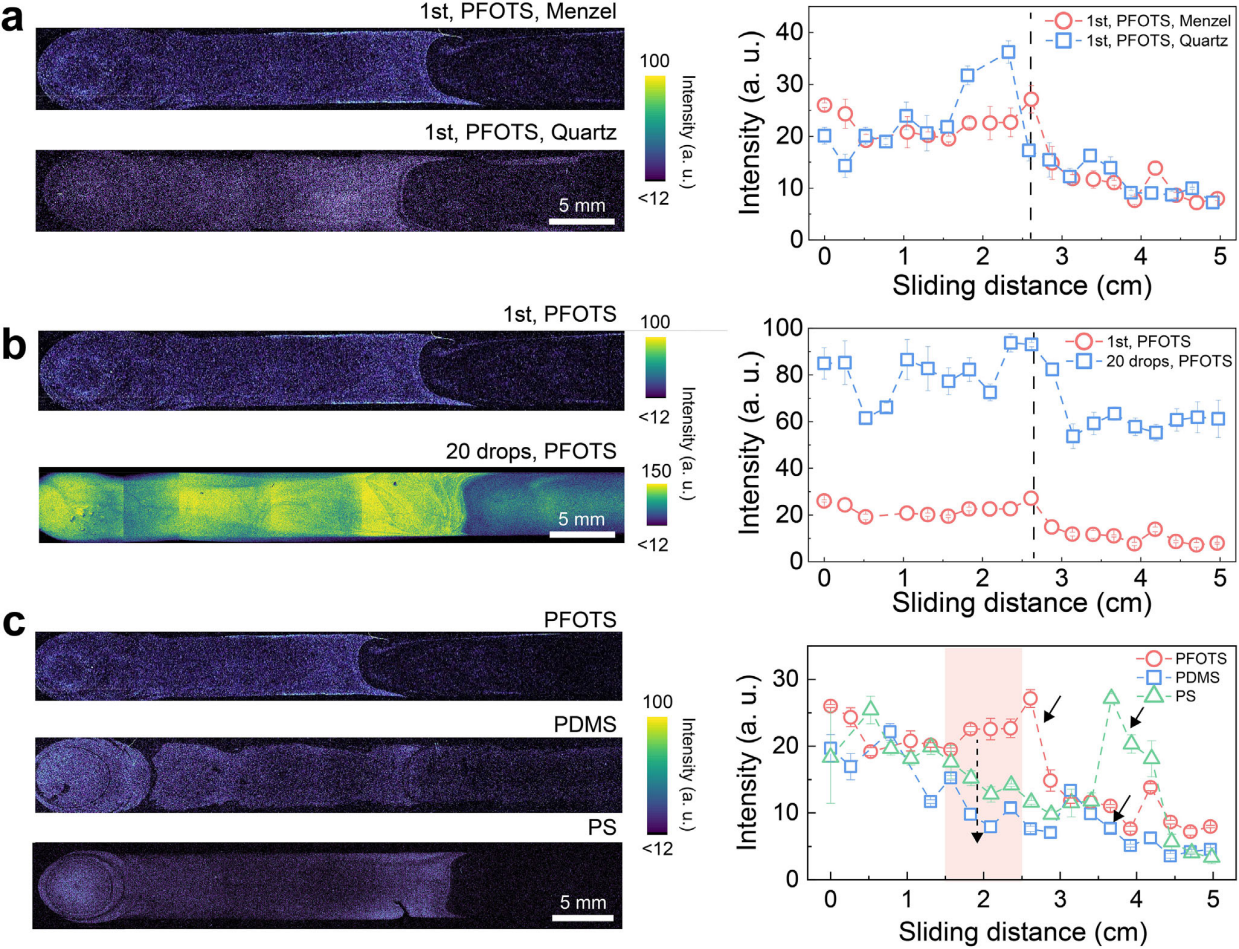
Universality of the deposition. a) Fluorescence images of PFOTS‐coated Menzel glass or quartz after sliding one PDI^+^ drop down the surfaces (left) and the corresponding average fluorescence intensity change with the sliding distance (right). b) Fluorescence images of PFOTS‐coated Menzel glass after sliding one or 20 drops down the surfaces (left) and the corresponding average fluorescence intensity versus sliding distance (right). c) Comparison of PDI^+^ deposition on the drop moving path on three different surfaces. In each case, the PDI^+^ concentration in the water drop was 500 nm.

The Menzel glass used as a substrate for the PFOTS or PS coatings in our experiments is a commercial sodium silicate glass, which contains some metal ions.^[^ ^40^ ^]^ In order to check if such ions affect the PDI^+^ deposition from sliding drops, we also performed experiments using quartz substrates. Similar deposition patterns were observed on Menzel glass and quartz substrates (**Figure** **4**a): the density of dye on the surface was highest at the beginning of the sliding path, then it decreased. After ≈2.5 cm an abrupt decrease was often observed, sometimes preceded by a transient sharp increase leading to a peak. On quartz substrates slightly higher average fluorescence intensity (deposited dye concentration) was observed after 2 cm. This is consistent with earlier studies showing that the negative surface charges resulting from slide electrification are more stable on quartz substrates.^[^ ^40^ ^]^

When multiple drops slide down a surface to repeat the process, the density of deposited PDI^+^ increases. After sliding 20 drops with PDI^+^ solution down a non‐charged surface, the deposition tendency was similar to that obtained after the first drop, but the amount of deposited dye increased with every drop (Figure 4b). The increase of the average fluorescence intensity was 3 times, but not 20 times. This is to be expected because in slide electrification the charge deposition efficiency decreases with the drop number due to a saturation process.^[^ ^10^ ^]^ The transition in the recorded average fluorescence intensity pattern ≈2.5 cm appears smoother than for a single drop. We can also create different homogenous deposition patterns on the surface by sliding several drops in either a cross or parallel direction (Figure S8, Supporting Information).

To analyze any possible influence of the specific surface chemistry,^[^ ^6^ ^]^ we compared the deposition on three different coatings including, 5 nm PDMS,^[^ ^41^ ^]^ 20 nm PS^[^ ^6^ ^]^ and 3 nm PFOTS,^[^ ^40^ ^]^ after the sliding of one water drop containing 0.5 µM PDI^+^ (Figure 4c). On PFOTS we observed the highest deposition, which correlates with the highest drop charge measured with pure water (+1.4 ± 0.2 nC).^[^ ^6^, ^9^ ^]^ For PS we measured 0.9 ± 0.2 nC and for PDMS we obtained a drop charge of 0.8 ± 0.3 nC. A lower amount and a smoother decrease of the PDI^+^ deposition with the sliding distance was detected on the PDMS surface. On PS, slightly more PDI^+^ was deposited than on PDMS and the distribution was less homogeneous. The sharp decrease in the deposition also occurs at different distances for different coatings (black arrows in Figure 4c). Overall, dye deposition decreases with slide distance on all surfaces.

The general pattern of the deposition process could also be demonstrated with another positively charged dye molecule, Rhodamine B (RhB) (Figure S9, Supporting Information). Deposition of RhB is also highly reduced when we add a gold layer underneath the PS coating. Since RhB also acquires several negative charges in its main structure, its deposition will be more complex, and we did not focus on this special mechanism here.

**Figure 5:**
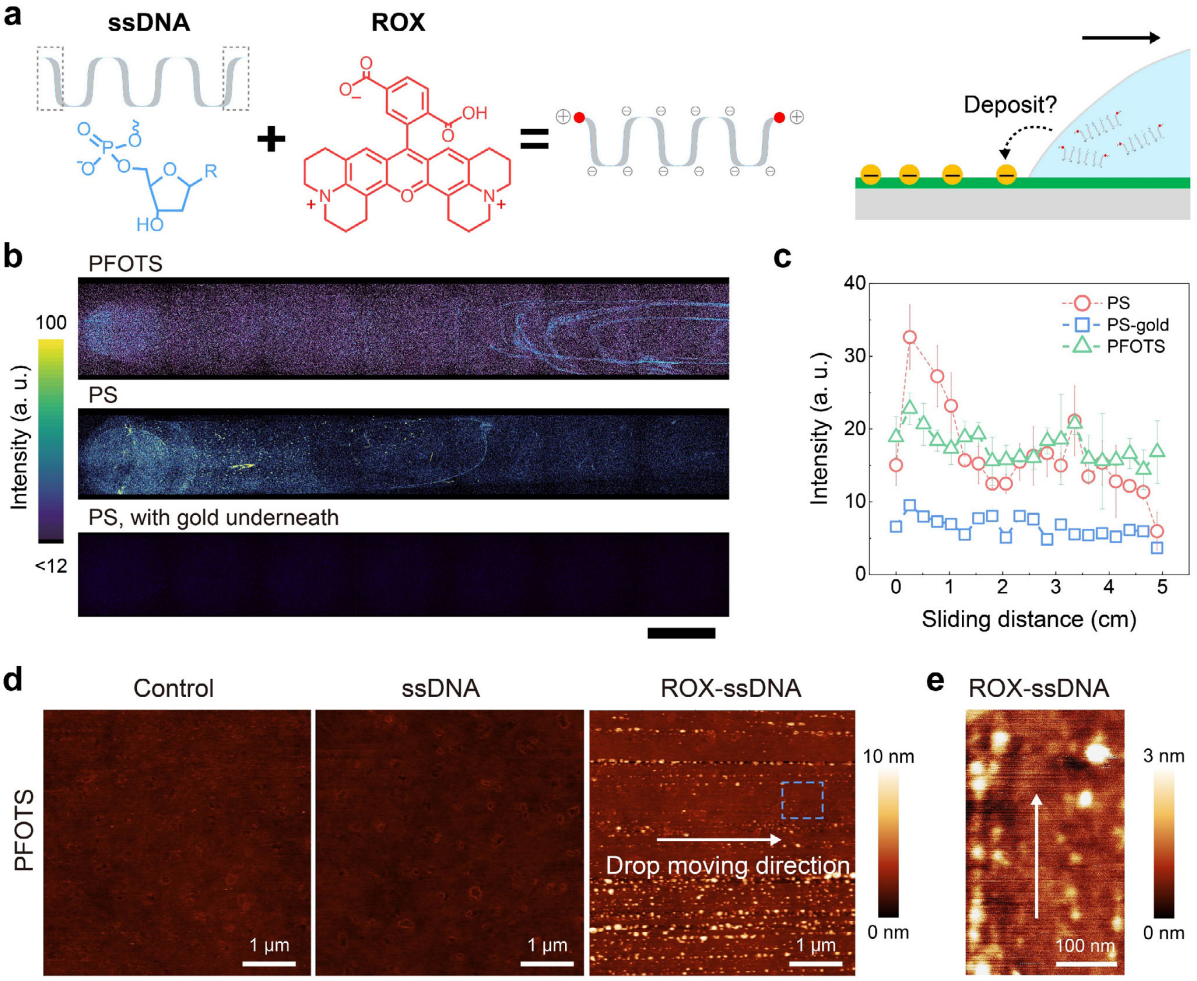
Deposition of the fluorescent single‐stranded DNA. a) The chemical structure of the end of a single‐stranded DNA (ssDNA), the chemical structure of ROX and a schematic of the ssDNA with attached positively charged ROX ends. b) Drop path after sliding a drop with 1 µm ROX‐ssDNA over the PFOTS coated Menzel glass and PS coatings without or with gold underneath on Menzel glass. Scale bar is 5 mm. c) The corresponding average fluorescent intensity versus sliding distance in (b). d) Scanning force microscopy images of the PFOTS‐coated surface after sliding of one pure water drop (control experiment), one water drop containing 1 µm of ssDNA and one water drop containing 1 µM of ROX‐ssDNA. e) Zoom‐in view in the blue frame in (d) illustrating the distribution of ROX‐ssDNA in a sparse area for PFOTS sample. Arrow lines illustrate the drop moving directions.

The deposition of large‐size biomolecules onto solid surfaces is crucial for applications such as biosensing and sequencing. For this reason, we also studied the influence of the spontaneous charging during drop sliding on the deposition of fluorescent biomolecules. As a model, we used carboxyrhodamine (ROX) labeled single‐stranded DNA (ROX‐ssDNA). ssDNA or RNA molecules, labeled with ROX are commercially available and broadly used in biological experiments.^[^ ^42^ ^]^ Such DNA or RNA molecules, fluorescently labeled with ROX are commercially available and widely used in biological experiments. In general, DNA molecules are negatively charged at neutral pH, which may hinder their adsorption to hydrophobic surfaces like polystyrene.^[^ ^43^ ^]^ However, the positively charged ROX is bound to the end of the DNA chain (**Figure** **5**a) and may promote deposition.

By sliding a water drop with 1 µm ROX‐ssDNA down a titled surface, we found a strong deposition on PFOTS and PS surfaces (Figure 5b). The patterns of intensity variation according to sliding distance are similar to those observed for PDI^+^ (Figure 2). However, the decrease in deposition with the sliding distance is less pronounced illustrating a lower energy barrier for this deposition (Figure 5c). Furthermore, on PS surfaces we detected a few points of high fluorescence intensity, indicating aggregation. When electrostatic effects were shielded by placing a gold layer beneath the PS film, the deposition of ROX‐ssDNA was reduced substantially (Figure 5b,c). These observations indicate that the positively charged ends of the ROX‐ssDNA chain are crucial for its deposition as reported before,^[^ ^44^ ^]^ because the positive charges at the ends improve the chance for this biomolecule deposition because of the slide electrification.

To verify that these fluorescent signals result from DNA deposition, we imaged the surfaces using scanning force microscopy (SFM) on PFOTS surfaces. The DNA molecules had a molecular weight of≈10 kg mol^−1^. Since the PFOTS surface is mechanically stable for force scanning and had a low roughness of RMS ≈ 0.5 nm, DNA could be identified. Figure 5d shows a scan of the middle area of the drop path. As a control, pure water drops were dropped onto the surface and the surface was imaged after the drop had slid off. After sliding a water drop across the pristine PFOTS surface, it appeared flat and featureless, showing no discernible difference compared to pure Menzel glass (Figure 5d). No significant deposition was detected after sliding a water drop with ssDNA without fluorescent‐labeling. After a drop with 1 µm ROX‐ssDNA has slid over the surface, ordered line‐shaped patterns of globular objects could be observed along the drop's moving direction.

The deposited globular objects from the drop had a height of 3–10 nm and a diameter of 5–20 nm, varying from single structures to aggregated ones (Figure 5e). These are most likely collapsed ROX‐ssDNA chains, which, unlike double‐stranded DNA, form random coils. The average spacing distance d between deposited ROX‐ssDNA can be roughly estimated by:

(3) $$d=\sqrt{A/n_{r}}$$

where A is the scanning area and n_r_ is the number of ROX‐ssDNA globular spots, assuming an even distribution. The individual DNA spots deposited on 5×5 µm^2^ PFOTS surface, counted as between 1800 and 2100, resulted in a mean spacing distance of ≈110 nm. This spacing is of the same order of magnitude as our calculations in previous sections.

## 3 Conclusion

As water drops slide on hydrophobic surfaces, they acquire a positive charge and leave negative charges (*primary ions*) on their path in a process called slide electrification. We found that the deposition of larger, *secondary* charged solutes, dissolved in water is substantially influenced by the process of slide electrification. For solutes with a charge *opposite* to the drop charge, deposition is hindered. For solutes with a charge similar to the drop charge, deposition is enhanced. In this case, the charge of the dissolved *secondary solute ions* is opposite to the charge of the *primary ions* left on the surface behind the sliding drop.

We propose that the *secondary* charged solutes are deposited at the rear contact line, as a result of three observations: 1) The *secondary* charged solutes are primarily deposited in the first 2–3 cm of sliding, which correlates with the deposition of *primary ions*. 2) When grounding the drop, the deposition of *secondary ions* is higher and does not decrease with slide distance. 3) Deposition is absent on conducting substrates.

This effect also applies to complex biomolecules, such as ROX‐ssDNA with cationically modified ends. The deposition of ROX‐ssDNA is enhanced deposition compared to situations in which the ends of ssDNA are not positively charged, or the slide electrification is prevented. We believe that these findings can provide valuable insights into understanding energy and mass transfer processes in both laboratory and industrial settings as well as biological processes. It can also contribute to the low‐cost fabrication or patterning of bio‐arrays for chemical or biological analysis.

## 4 Experimental Section

### Surface Preparation

Perfluorodecyltrichlorosilane (PFOTS) layers were prepared by chemical vapor deposition (CVD). The glass substrate (Epredia Menzel or Sigma‐Aldrich Quartz, size: 76 ×26 mm^2^, thickness: 1.0 mm) was treated with O_2_‐plasma at 100 W for 10 min (Femto low‐pressure plasma system, Diener electronic). The surface was then placed in a vacuum desiccator containing a dish with 1H,1H,2H,2H‐perfluorooctadecyltrichlorosilane (97%; Sigma‐Aldrich). The desiccator was evacuated to 80–100 mbar and then closed. The reaction was allowed to proceed for 2 h. Finally, the surfaces were rinsed with ethanol to remove unbound silanes. The static advancing contact angle Θ_A_ and receding contact angle Θ_R_ of 10 µL water drop on fluorinated surfaces were 117° ± 2° and 96° ± 2°, respectively. Polydimethylsiloxane (PDMS) coated surfaces were prepared by placing the O_2_‐plasma activated glass slides in 100 mL of silicon oil (100 cSt, molecular weight *M_w_* = 6000 g mol^−1^, Acros Organics) at 150 °C for 24 h. The glass slides were cleaned consecutively in hexane and alcohol with sonication for 3 min and rinsed with toluene three times. Θ_A_ and Θ_R_ of water on PDMS surfaces were 103° ± 1° and 92° ± 1°, respectively. Polystyrene (PS) films were prepared by dip‐coating gold‐coated glass slides into 1 wt.% toluene solution (*M_w_* = 192 kg mol^−1^, Sigma–Aldrich). Gold layers were prepared by sputtering 35 nm of gold and 2 nm of an adhesive chromium layer onto glass slides. After moving the substrates at a speed of 90 mm/min into the solution and waiting for 10 s, the substrates were moved up again at 10 mm min^−1^. Finally, the films were annealed at 120 °C under vacuum for 24 h. The PS films were 20 nm thick, as measured by a profiler (KLA‐Tencor). Θ_A_ and Θ_R_ of water on PS surfaces were 95° ± 2° and 88° ± 2°, respectively. PFOTS coated glass samples showed a two‐fold higher charging of drops and substrate than PDMS and PS coated surfaces.^[^ ^6^ ^]^

### Fluorescent Molecules

Fluorescent dyes N,N′‐bis(2,6‐diisopropylphenyl)‐1,6,7,12‐tetra‐[3‐(N‐methylpyridinium)oxy] perylene‐3,4,9,10‐tetracarboxylic acid diimide iodide (PDI^+^) and N,N′‐bis(2,6‐diisopropylphenyl)‐1,6,7,12‐tetra‐[(4‐sulfonyl)phenoxy] perylene‐3,4,9,10‐tetracarboxylic acid diimide sodium (PDI^−^) was synthesized as reported before.^[^ ^30^ ^]^ Rhodamine B (RhB) was purchased from Merck KGaA, Germany. Single‐stranded DNA (ssDNA) was purchased from biomers.net GmbH, Germany, and fluorescent single‐stranded DNA (ROX‐ssDNA) was fabricated by BOC Sciences, USA. Their base sequences were: 5′‐ROX‐ CCTGCTTCTATTTGTCTTGCAGTAACACGCCA‐3′. The molecule weight is≈9700 g mol^−1^.

### Drop Sliding

The drop sliding experiments were conducted on a tilted platform^[^ ^6^ ^]^ at 40° (Figure 1). A 45 µL drop of Milli‐Q water with solutes was deposited at the top of the titled hydrophobic surface by a grounded syringe needle. The needle was connected to a peristaltic pump (MINIPULS 3, Gilson). The experiments were conducted at a temperature of 25 ± 0.5 °C and a humidity of 30 ± 5% to minimize environmental effects. Before every experiment, the surface was neutralized by an ionizing air blower (Aerostat PC ionizing air blower, Simco‐Ion) with 100% power factor for 5 min directly blowing in 5 cm distance to make sure neglectable charge was left on the surface before the experiment. The drops fell from a height of ≈ 5 mm which was similar to the drop size itself to avoid drop re‐bouncing. The initial charge in the drop was neutralized by a grounded copper wire when it first contacted the surface. The setup was placed in a Faraday cage. Each sliding process is repeated on at least three different samples to ensure reproducibility. Drop charges can be determined after sliding 4 cm with an electrode connected to a current amplifier. The current was integrated over the first 2 ms. More details can be found in the references.^[^ ^6^, ^10^ ^]^

### Laser Scanning Confocal Microscopy (LSCM)

LSCM images were taken on an inverted confocal microscope (Leica TCS SP8 SMD) using the Leica LAS X software.^[^ ^45^ ^]^ Horizontal visualization (xy imaging) of the deposited ions on surfaces was obtained using an HC PL FLUOTAR 2.5×/0.07 CORR CS DRY objective. For PDI^+^, an argon laser line (514 nm) was used for excitation. The excitation light intensity can be controlled by an acousto‐optical tunable filter (AOTF) and transmission was set to 40%. The collected emission was detected in the wavelength range between 580 and 610 nm with a detector gain value set to 1250. For PDI^−^ ions, a DPSS laser (561 nm) was used. The AOTF transmission was set to 60%. The collected emission was detected in the wavelength range between 600 and 650 nm with a detector gain value set to 1250. The excitation light intensities adjusted by the AOTF transmission were chosen in such a way that similar fluorescence intensity can be detected from PDI+ and PDI‐ with similar concentrations (Section S2, Supporting Information). The confocal images were acquired along the trajectory of the droplet, each covering an area of 6.2 × 6.2 mm^2^. The scanning time was no more than 10 s to prevent photobleaching. Subsequent images were acquired by incrementing the y‐axis coordinate (representing the direction of droplet displacement) by 6 mm. Before capturing each image, it was necessary to re‐focus by adjusting the z‐axis value to make sure the solid‐air interface was also observedprecisely. These images were converted into a data matrix with pixel dimensions of 12 × 12 µm^2^ using a custom Python code. Details can be found in: https://github.com/longteng94/Confocal‐data‐analysis. The individual images were subsequently aligned at their edges and assembled sequentially to construct a comprehensive representation of fluorescent signals along the entire droplet trajectory. The intensities were analyzed by Gwyddion software.^[^ ^45^ ^]^

### Force Microscopy Measurements

Surface and DNA topography analysis were conducted in a wide area using Scanning Force Microscopy (SFM, Dimension Icon with NanoScope 6 controller, Bruker Corporation). The imaging was performed utilizing PeakForce Quantitative Nanomechanical Mapping (QNM) mode, with a scan rate of 0.96 Hz and a resolution of 1024 × 1024 pixels. For the measurements, a Brucker OLTESPA cantilever was selected, characterized by a nominal resonant frequency of *f* = 70 kHz and a spring constant of *k_z_* = 2 N m^−1^. The sensitivity is 140.37 nm V^−1^ after no‐ touch calibration. The setpoint force was 20 nN. The force scanning image was also measured by the JPK NanoWizard 4 AFM using the “Quantitative Imaging” (QI) with the same setting and cantilever for double check and zoom‐in view.

## Conflict of Interest

The authors declare no conflict of interest.

## Author Contributions

X.Z., K.K., and H.‐J.B. designed the experiment. X.Z. and K.K. performed the confocal measurements. J.G.L. and K.P. synthesized and analyzed fluorescent molecules. X.Z., Y.J., and H.B. prepared the DNA samples and did the SFM high‐resolution measurements. X.Z. and Z.N. did the surface preparation. X.Z., S.J., and H.‐J.B. analyzed the deposition process. R.B. and N.K. did the surface charge distribution measurements. All the authors wrote the manuscript and have approved the final version.

## Supporting information



Supporting Information

## Data Availability

The data that support the findings of this study are available from the corresponding author upon reasonable request.
