# Supplementary material for: Spontaneous Charging from Sliding Water Drops Determines the Interfacial Deposition of Charged Solutes
Source: Adv Mater. 2025 Mar 7;37(16):2420263. doi: 10.1002/adma.202420263 (PMC12016736; doi:10.1002/adma.202420263)
Supplement: Supplementary file 1 — Supporting Information [file ADMA-37-2420263-s001.docx]

**Supporting information**

**Spontaneous charging from sliding water drops determines the interfacial deposition of charged solutes**

Xiaoteng Zhou,^1,#^ Yuwen Ji,^1^ Zhongyuan Ni,^1^ Javier Garcia Lopez,^2^ Kalina Peneva,^2,3,4^ Shan Jiang,^5^ Nikolaus Knorr,^1^ Rüdiger Berger,^1^ Kaloian Koynov,^1,^* Hans-Jürgen Butt^1,^*

1. Max Planck Institute for Polymer Research, Ackermannweg 10, 55128, Mainz, Germany.

2. Institute of Organic Chemistry and Macromolecular Chemistry, Friedrich Schiller University Jena, Lessingstraße 8, 07743 Jena, Germany.

3. Center for Energy and Environmental Chemistry Jena (CEEC Jena) Friedrich Schiller University Jena, Philosophenweg 7a, Jena, 07743, Germany,

4. Jena Center for Soft Matter (JCSM), Friedrich Schiller University Jena, Philosophenweg 7a, Jena, 07743, Germany

5. Department of Materials Science and Engineering, Iowa State University of Science and Technology, Ames, IA 50011, USA.

*Email: [koynov@mpip-mainz.mpg.de](mailto:koynov@mpip-mainz.mpg.de), [butt@mpip-mainz.mpg.de](mailto:butt@mpip-mainz.mpg.de),.

^#^Present address: Massachusetts Institute of Technology, 77 Massachusetts Avenue, Cambridge, Massachusetts, 02139, USA

Keywords: slide electrification, interfacial phenomena, mass transfer, wetting, ssDNA

**Supplementary Section 1. Kelvin probe method to measure the charge distribution on a hydrophobic surface.**

**Sample preparation**

A 170 µm thick glass slides (Precission cover glass, Marienfeld, Germany) with dimensions of 60×24 mm^2^ were coated with a 20 nm thick polystyrene (PS) film from solution via dip coating. The coated glass slides were then bonded to indium tin oxide (ITO) coated glass slides (Sigma-Aldrich) in standard microscopy slide format (75 ×25 ×1 mm^3^) using double-sided conductive adhesive tape (1182 Conductive Metallic Tape, 3M).

Charging was performed by sliding fifty 45 µL drops of pure water at a tilt angle of 40°. Both the steel capillary used for dispensing the drops and the ITO back electrode of the sample were connected to ground during the drop sliding process. The height of the capillary above the sample was adjusted so that the drop would detach purely by gravity just before contacting the sample, thus avoiding excessive splashing upon impact. To prevent charging of the drop from the initial impact, a thin grounded tungsten wire was positioned from above, contacting the drop after approximately 1 mm of sliding.

**Kelvin probe method**

Surface potential maps were recorded using an RHC020 device from KP Technology,^[1]^ which is equipped with a steel electrode probe with a bottom diameter of 0.5 mm (Figure S1a). The samples were grounded with a metal clamp that made contact with the ITO layer. In the classical Kelvin probe method, the surface potential ($U_{SP}$) is determined by measuring the induced AC voltage between the oscillating probe and the back electrode of the charged sample while applying a "backing" DC voltage ($V_{b}$) via a feedback loop to the sample.^[1]^ When the applied DC voltage reaches $U_{SP}$, the electric field between sample and the probe surface vanishes. The RHC020 device employs an "off-null" detection method, where $U_{SP}$ is determined by linear extrapolation of measurements at varying $V_{b}$, instead of relying on nulling.^[2]^

**Surface charge density calculation**

The surface charge density$\sigma\left( x,y \right)$ of the charges that are located on the surface of the insulating film can be approximated by the capacitor equation:^[3]^

$\text{σ}\left( \text{x,y} \right)\text{ }\text{=}\text{ }\text{ε}\text{ε}_{\text{0}}\frac{\text{U}_{\text{SP}}\left( \text{x,y} \right)}{\text{d}}$, (S1)

where, $\text{ε}$ is the dielectric constant of the glass slide, $\text{d}$ the thickness of the glass slide, and $\text{ε}_{\text{0}}$ the electric permittivity of free space. The measured charge density is an average over the area corresponding to the probe size.

**Results**

The color scale of the surface charge density ranges from 0 (blue) to -0.2 nC/cm^2^ (red), as calculated by Equation S1. The negative charge was generated by the 50 drops sliding down the PS surface. Sliding drops separate negative charge from the drop, while the drops themselves become positively charged. Surface charge densities of slid drops on polymer thin films have also been previously reported^[4]^ and $\text{U}_{\text{SP}}\left( \text{x,y} \right)$ was recorded using electrostatic voltmeters. Knorr et al imaged the charge distribution using mixed toner powders.^[5]^ While the powder method is destructive, both the Kelvin probe and electrostatic voltmeter methods are non-contact and non-destructive.

**
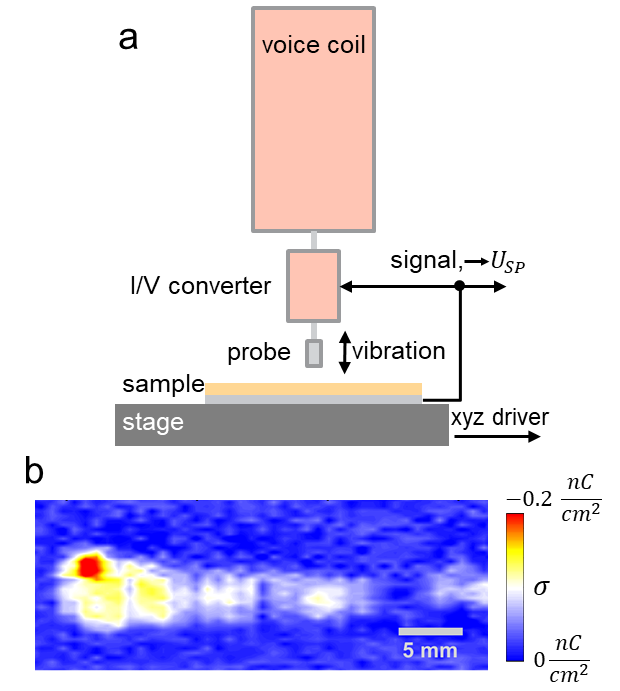
**

**Supplementary Figure S1.** (a) The setup using Kelvin probe method to measure the charge distribution. (b) Charge distribution map shown by charge density difference.

**Supplementary Section 2. Optimizing the experimental conditions for the laser scanning confocal microscopy (LSCM) imaging.**

While the PDI^+^ and PDI^─^ have similar core structures, their absorption and emission spectra differ substantially.^[6]^ Both dyes were excited with different lasers, namely the 514 nm line of an Argon laser for PDI^+^ and the 561 nm line of an DPPS laser for PDI^─^. As the LSCM experiments were used to estimate and compare the amounts of deposited dyes, under optimal conditions the same fluorescence intensities should be obtained from PDI^+^ and PDI^─^ deposit at equal surface densities. To achieve such equal surface densities, we let 10 µL water drops containing either PDI^+^ or PDI^─^, at equal volume concentrations (500 nM) fully evaporate on PFOTS coated glass slides. Figure S2 shows the dependence of the fluorescence intensities recorded on these surfaces versus the corresponding excitation light intensity. The excitation intensities were tuned by controlling the transmission of an acousto-optical tunable filter (AOTF) and are shown in units of % transmission compared to the maximum laser intensity. As can be seen (Figure S2) the detected fluorescence signal from both dyes increases almost linearly with the excitation intensity. Here we did not increase the AOTF transmission for the 561 nm line (PDI^─^) more than 60% in order to avoid photobleaching. One can see that similar fluorescent intensities can be observed for about 30% transmission of the 514 nm line (PDI^+^) and 60% transmission of the 561 line (PDI^─^). However, as direct comparison of the fluorescence signals of the two dyes is performed only for the experiments illustrated in Figure 2b in the main text and all other experiments were performed with PDI^+^, in all further LSCM experiments we set the transmissions to 40% for the 514 line (PDI^+^) and to 60% for the 561 line (PDI^─^). These settings ensured strong enough fluorescence signal from the PDI^+^.

LSCM images recorded under these settings for PDI^+^ and PDI^─^ deposit at equal surface densities are shown in Figure S3. The obtained fluorescence intensity for PDI^+^ is 20 % to 25 % higher than that of PDI^─^ (also Figure S2). Therefore, when we compared the fluorescence intensities recorded for the dyes deposit from sliding droplets (Figure 2c), the data for PDI^─^ were multiplied with a correction factor of 1.25.


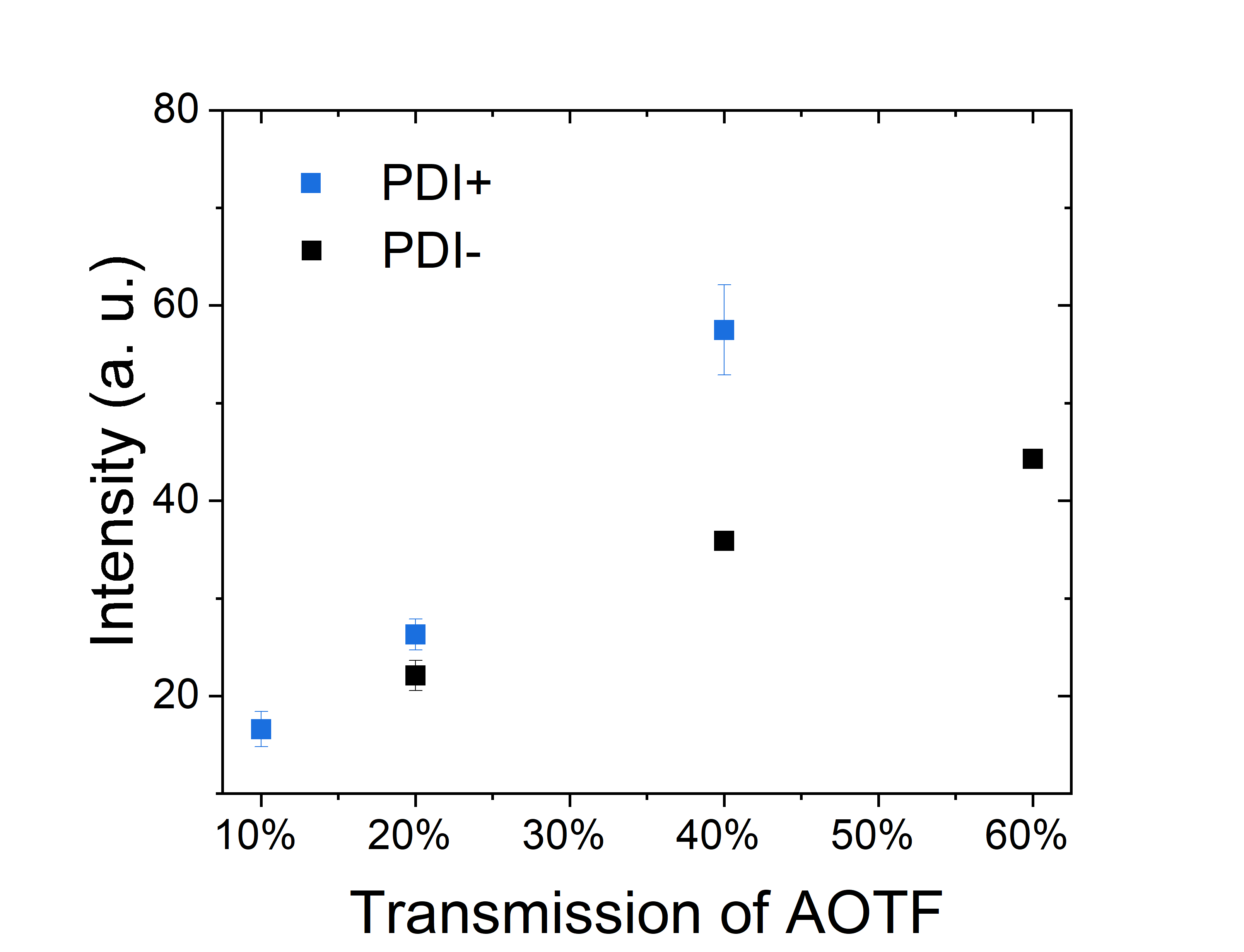


**Supplementary Figure S2.** Fluorescence intensities recorded for PDI^+^ and PDI^─^ molecules deposit at equal surface densities on PFOTS surfaces versus the excitation laser light intensities controlled by the transmission of the AOTF.


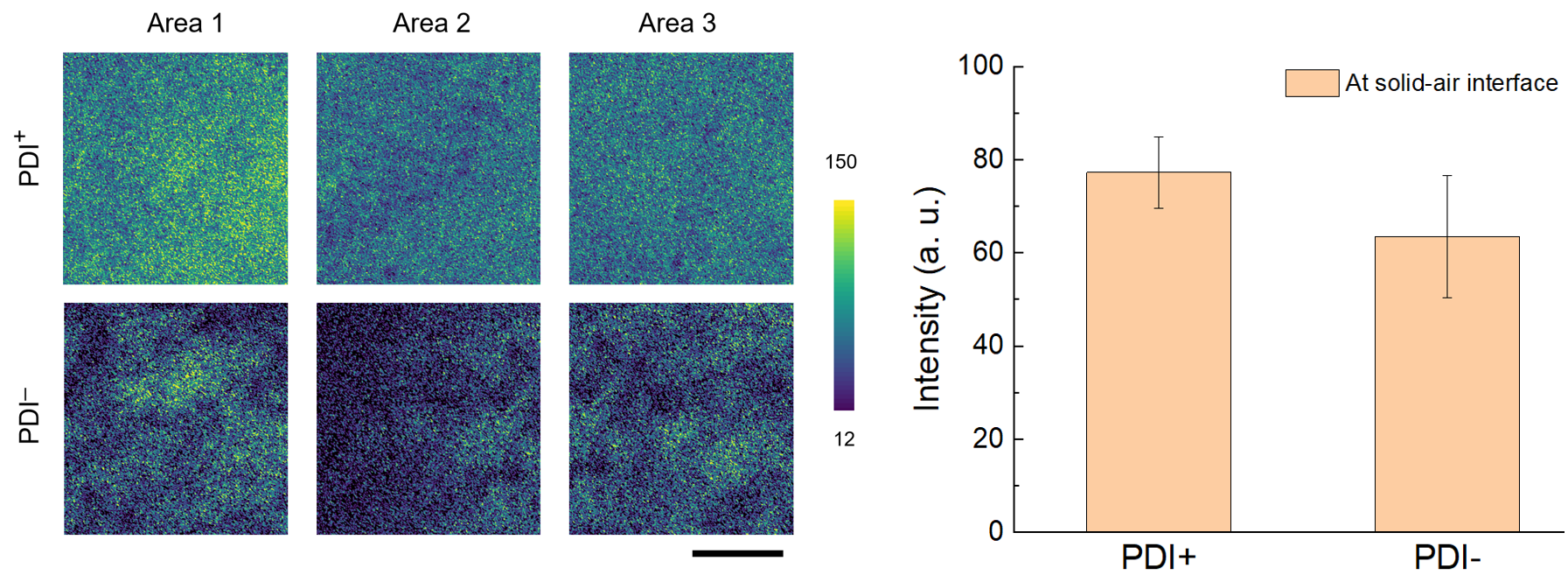


**Supplementary Figure S3.** LSCM images recorded at three different areas below water drops containing 500 nM PDI^+^ or PDI^-^ after the drops fully evaporated on PFOTS surfaces. These areas are chosen between the center and the initial edge to prevent high aggregation and non-uniform distribution. The AOTF transmission was set to 40% for the 514 nm excitation (PDI^+^) and to 60% for the 561 nm excitation (PDI^─^). Scale bar: 1 mm.

**Supplementary Section 3. Humidity effects on PDI^+^ deposition**

To evaluate the effect of humidity on deposition of charged solutes we carried out experiments at 10% and 80% humidity. The experiments were done inside a closed chamber. Humidity was controlled by mixing a flow of nitrogen which was saturated before by letting it bubble through water, and dry nitrogen. The value is confirmed by a humidity sensor fabricated inside the chamber. Thus, the background gas was nitrogen.





**Supplementary Figure S4**. Deposition at low and high humidity. (a) The setup to change the environment humidity during drop sliding. The humidity in the closed measuring cell was adjusted by mixing 100% humid nitrogen with dry nitrogen. In these experiments we used PFOTS coated on glass substrate. Titling angle is 40°. (b) Confocal microscope images of a PFOTS surfaces after sliding a 45 µL drop of water containing 500 nM PDI^+^ at 10 % and 80 % humidity. (c) Fluorescence intensities versus slide length along the dashed lines.

**Supplementary Section 4. PDI^+^ deposited on a PFOTS surface with a metal interlayer**

To demonstrate that the dielectric permittivity or conductivity influenced deposition, a metal interlayer between PFOTS coating and glass was fabricated by a sputtering 1 nm Chromium (Cr) as an adhesive layer and 5 nm Aluminum (Al) which can be oxidized in air quite easily underneath the coating. The oxidized Al has sufficient reactivity to chemical bind PFOTS. Then we can have a conductive (or high permittivity) shield part in both the middle area (case i in Figure S5) and the end of the substrate (case ii in Figure S5) to compare the difference of the deposition.


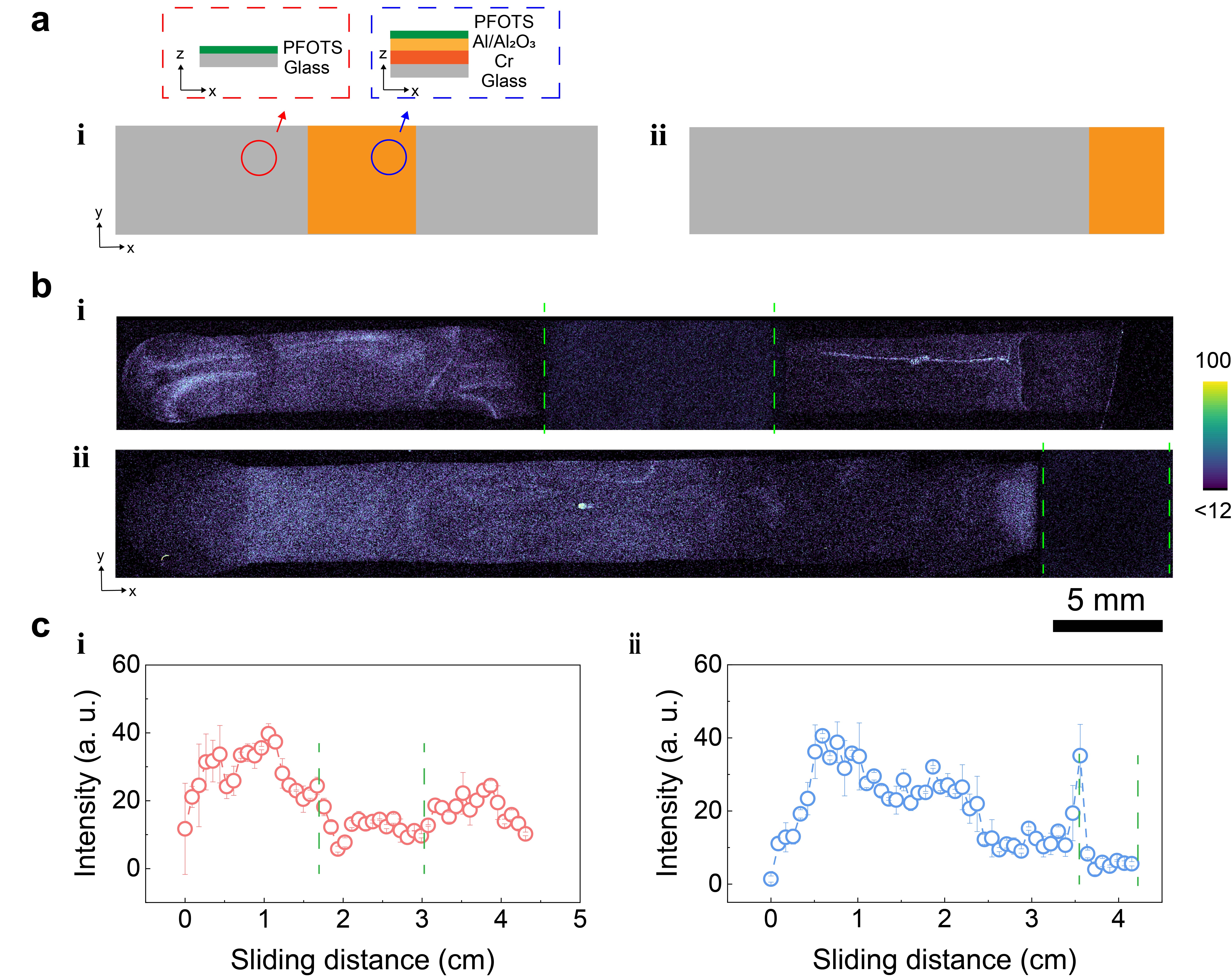


**Supplementary Figure S5**. (a) Schematic of the coating of the two test samples. (b) Confocal microscope images of the deposited PDI^+^ after sliding a drop of 45 µL water containing 500 nM PDI^+^ down a sample tilted by 40°. (c) Mean fluorescence intensity along the sliding path for a sample fabricated with an interlayer in the middle area of substrate (i) and in the end part of the substrate (ii).

**Supplementary Section 5. PDI^+^ deposited on a pre-charged surface**

To see the effect of pre-charging, we let 10 pure water drops slid down the PFOTS surface to pre-charge it. Then we let a water drop containing 500 nM PDI^+^ slide over the surface. Afterwards, we characterized the sliding path by confocal microscopy to visualize the deposition (Figure S6). Deposition looked even more inhomogeneous than on the neutral surface and the total amount adsorbed slightly increased.


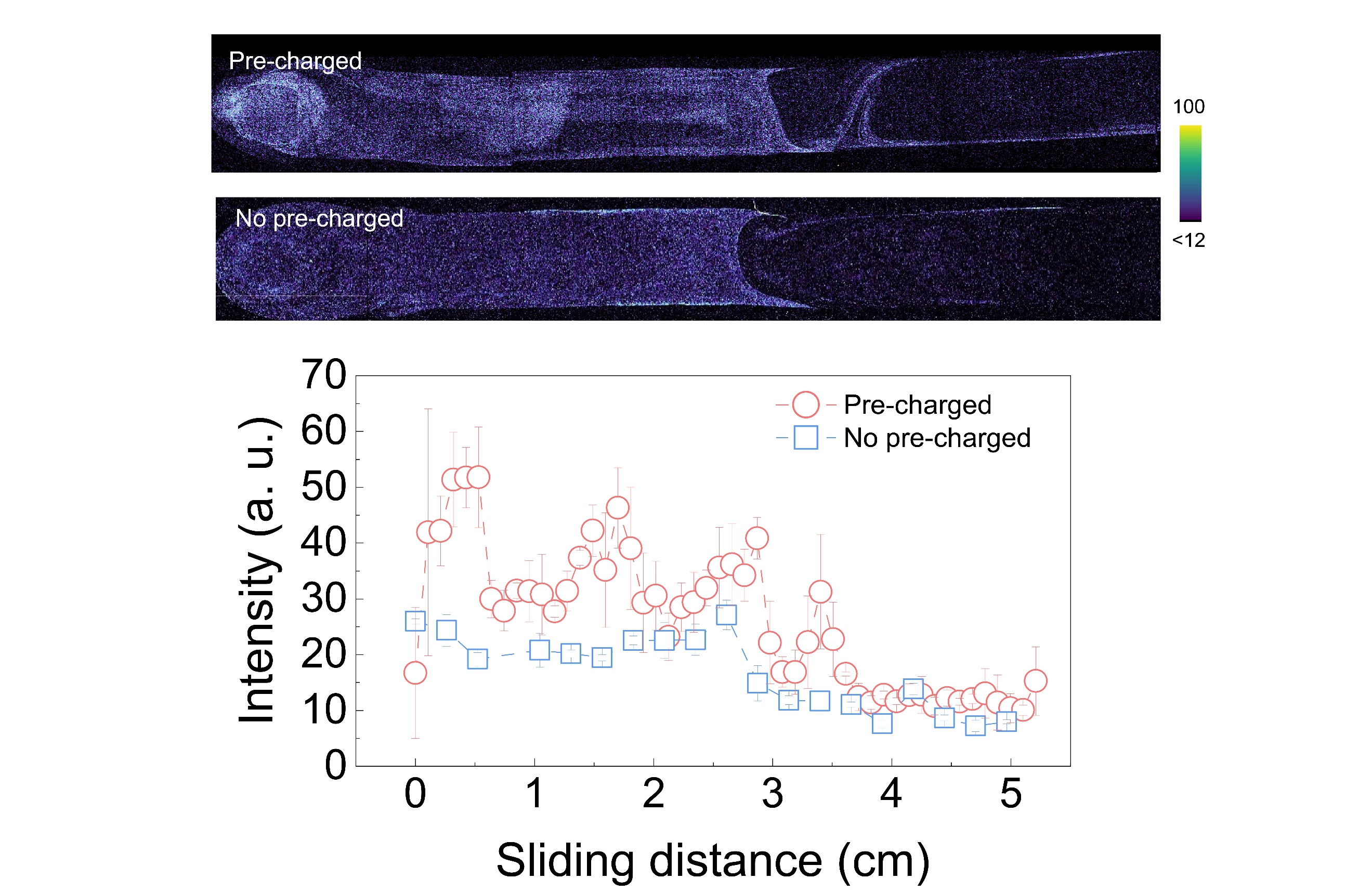


**Supplementary Figure S6**. Comparison of solute deposition after one water drop with 500 nM PDI^+^ slides over a 10 pure water drop pre-charged surface to a charge-free surface.

**Supplementary Section 6. Fluorescence correlation spectroscopy (FCS) estimation of the deposited PDI density.**

Fluorescence Correlation Spectroscopy experiments were performed on a commercial device, LSM 880 (Carl Zeiss, Jena, Germany). The excitation was done with the 514 nm line of an Argon laser focused into the studied samples through a C-Apochromat 40×/1.2 W water immersion objective (Carl Zeiss, Jena, Germany). The emission light was collected with the same objective and after passing through a confocal pinhole, directed to a spectral detection unit (Quasar, Carl Zeiss) in which a detection range of 580 – 610 nm was selected.

First, aqueous solutions of the PDI^+^ and a reference dye, rhodamine 6G (Rh6G) were studied in order to estimate the hydrodynamic radius and the fluorescence molecular brightness of the PDI^+^. The solutions were placed in eight-well polystyrene chambered cover glass (Nunc™ Lab-Tek™, Thermo Fisher Scientific, Waltham, MA, USA). The confocal observation volume was positioned in the solution, about 100 µm above the bottom of the chamber and series of FCS measurements with a total duration of 150 s were performed. The time-dependent fluctuations of the fluorescent intensity δI(τ) were recorded and analyzed by an autocorrelation function *G*(τ) = 1 + <δI(t) · δI(t + τ) >/<I(t)>^2^. The obtained experimental autocorrelation curves were fitted with a theoretical model function^[7]^ for bulk 3D diffusion:

$G(\tau)=1+\left[ 1+\frac{f_{T}}{1-f_{T}}e^{-\tau/\tau_{T}} \right]\frac{1}{N}\frac{1}{\left[ 1+\frac{\tau}{\tau_{D}} \right]\sqrt{1+\frac{\tau}{S^{2}\tau_{D}}}}$ (S2)

Here, *N* is the average number of fluorescence species in the observation volume, *f_T_* and *τ_T_* are the fraction and the decay time of the triplet state, *τ_D_* is the diffusion time of the fluorescent species and *S* is the so-called structure parameter, *S = z_0_/r_0_*, where *z*_0_ and *r*_0_ represent the axial and radial dimensions of the confocal volume, respectively. Furthermore, the diffusion time, *τ_D_*, is related to the diffusion coefficient, *D*, through: $\tau_{Di}=\frac{r_{0}^{2}}{4Di}$. The fits yielded the corresponding diffusion times, and subsequently the diffusion coefficients and trough the Stokes-Einstein relation the hydrodynamic radius (R_h_) of the fluorescent species. Finally, the fluorescence molecular brightness of the fluorescence molecules was estimated as FB = <I(t)>/*N.* As the value of *r*_0_ and *z*_0_ depend on the specific characteristics of the optical setup, calibration experiments were performed using fluorescent tracers with known diffusion coefficients, i.e. rhodamine 6G (Rh6G). We obtained *r*_0_ = 0.2 µm and *z*_0_ = 1.2 µm.

**
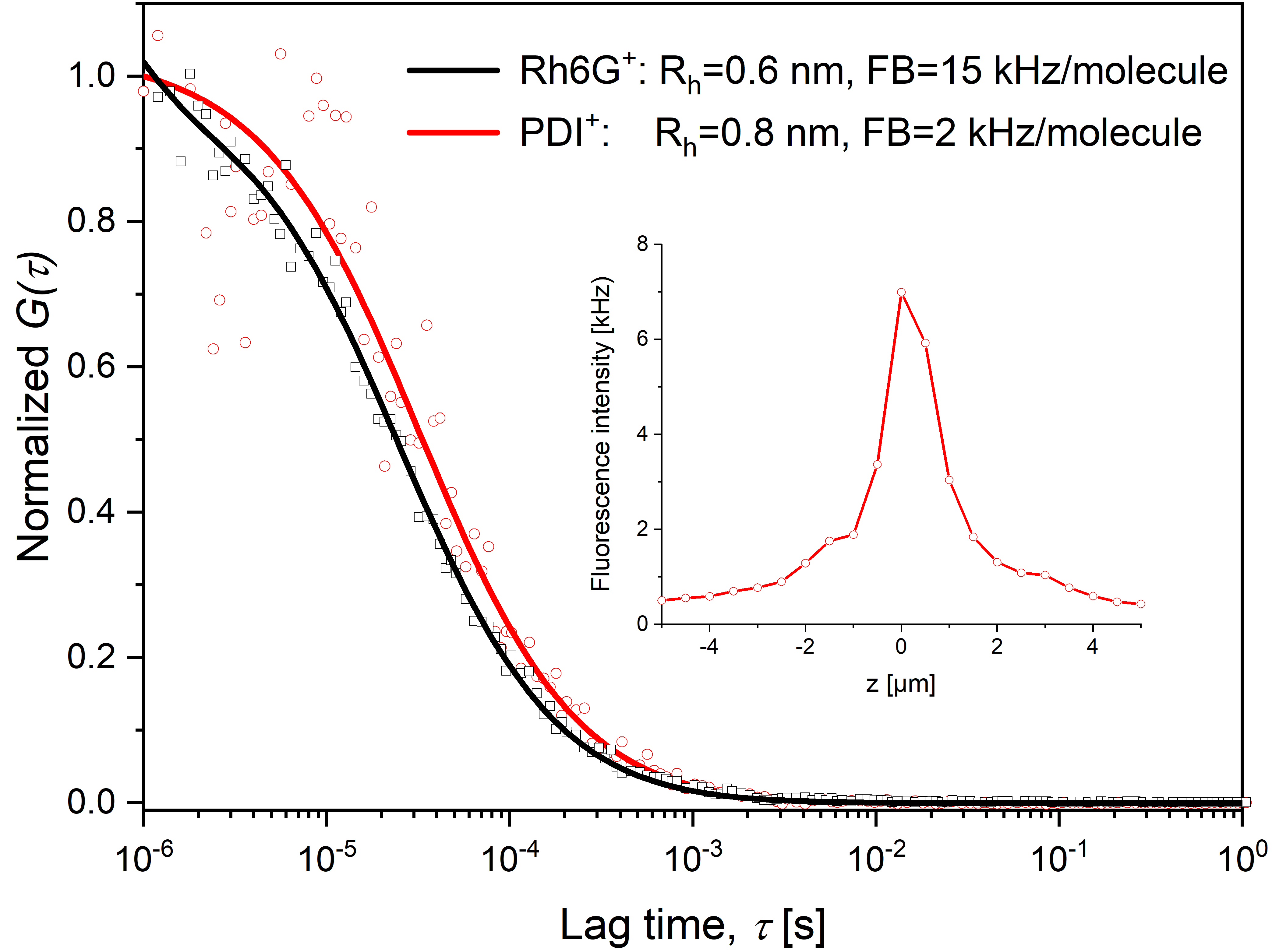
**

**Supplementary Figure S7.** FCS studies of the PDI^+^. Normalized autocorrelation curves (symbols) and the corresponding fits with eq. S2 (solid lines) for PDI^+^ and a reference dye (Rh6G) in aqueous solutions (10 nM). Inset: z-scan trough a PS surface with deposited PDI^+^.

The experimental autocorrelation curve measured for PDI^+^ is shown in Figure S7 together with the respective fit with eq. S2. The fit yielded values of *R_h_* = 0.8 nm for the hydrodynamic radius of the PDI^+^ molecules and *FB* = 2 kHz/molecule for their molecular brightness under the applied experimental conditions.

Next, we studied the density of the deposited PDI^+^ behind a droplet with initial concentration of 500 nM sliding on a glass slide coated with PS. The glass slide was mounted on the FCS device and a drop of water placed on the PS to make the deposited PDI re-diffuse. The focus was adjusted close to the beginning of the drop sliding path. The FCS confocal detection volume was scanned in a direction perpendicular to the PS surface to record so called “z-scan” that is the average fluorescent intensity vs. the focus position in normal direction under the same experimental condition as the bulk FCS experiments. Such z-scan is shown as an inset in Figure S7. A bell-shape curve was obtained reflecting the convolution of the confocal volume normal dimension with the monolayer of PDI^+^ molecules on the PS surface. By dividing the maximum fluorescent intensity at the top of the curve 7 kHz to the fluorescence brightness (FB = 2 kHz/molecule) of the individual PDI+ molecules were estimate that in average 3.5 molecules are present in the confocal volume on the surface of the PS layer. Considering that the cross-section of the confocal volume and the PS surface have an aria of π*r*_0_*^2^* = 0.13 µm^2^, we estimate a surface density of the deposited PDI^+^ molecule of about 2.7×10^13^ molecules/m^2^. This value corresponds to about 190 nm spacing between the PDI^+^ molecules at the surface and is very close to the ones estimated based on the drop charge measurement as described in the main manuscript.

**Supplementary Section 7. Writing patterns**

We let a 33 µL water drop with 500 nM PDI^+^ sliding down PFOTS surfaces at a tilting angle of 40° in designed ways. The sliding distance is 2 mm before the deposition starts to decrease. By sliding several drops in different positions, different writing patterns can be obtained as shown in Figure S8.


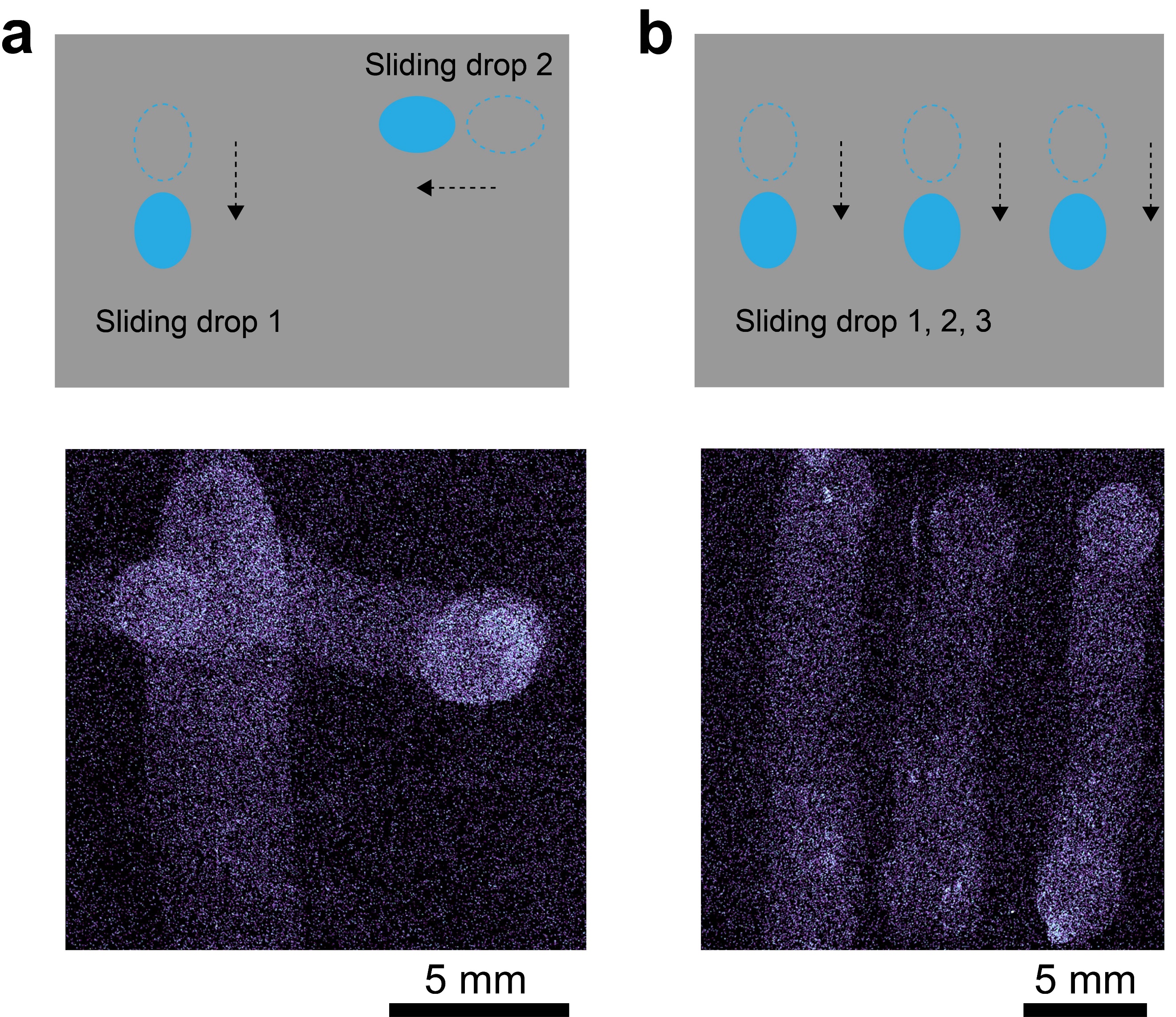


**Supplementary Figure S8.** Pattern writing by using 33 µL free drops with 500 nM PDI^+^ sliding down PFOTS surfaces at a tilting angle of 40° in different ways to create (a) a cross pattern or (b) a parallel pattern.


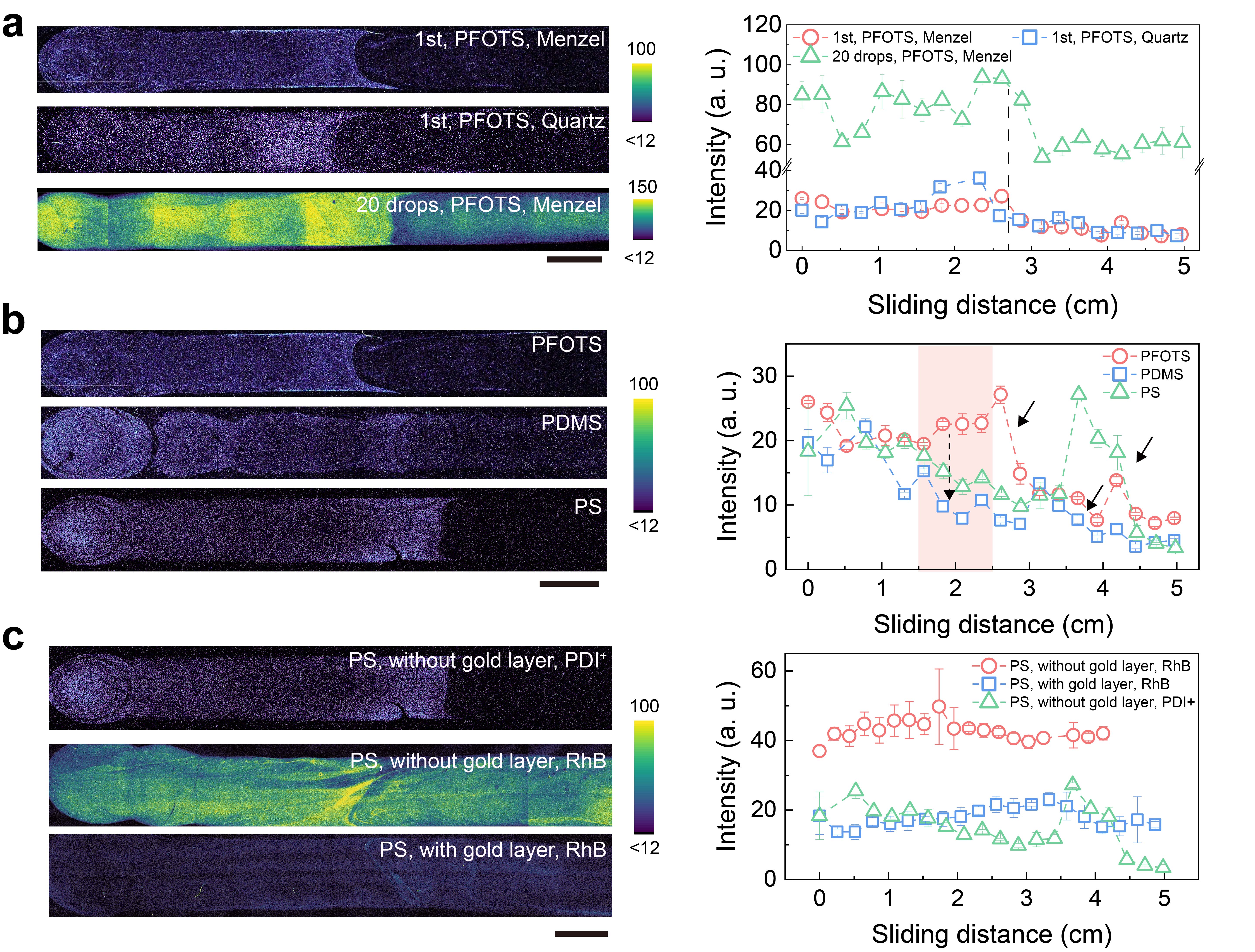


**Supplementary Figure S9.** Comparison of the PDI^+^ and RhB depositions on the PS surfaces. Titling angle: 40 °. Scale bar: 5 mm. For all these cases, the dye concentration was 500 nM in the water drop.

**References**

[1] C. W. Reedyk, M. M. Perlman, *Journal of the Electrochemical Society* **1968**, 115, 49.

[2] I. Baikie, P. Estrup, *Review of scientific instruments* **1998**, 69, 3902.

[3] a)J. Cross, **1987**; b)R. Gerhard-Multhaupt, in *Wiley Encyclopedia of Electrical and Electronics Engineering*.

[4] a)K. Yatsuzuka, Y. Mizuno, K. Asano, *Journal of Electrostatics* **1994**, 32, 157; b)B. He, A. A. Darhuber, *Journal of Micromechanics and Microengineering* **2019**, 29, 105002.

[5] N. Knorr, S. Rosselli, G. Nelles, *Langmuir* **2024**, 40, 14321.

[6] J. R. Lakowicz, *Principles of fluorescence spectroscopy*, Vol. 1, Springer, **2006**.

[7] R. Rigler, E. S. Elson, *Fluorescence correlation spectroscopy: theory and applications*, Vol. 65, Springer Science & Business Media, **2012**.
